# Inaccuracies in survey reporting of alcohol consumption

**DOI:** 10.1186/s12889-019-7987-3

**Published:** 2019-12-05

**Authors:** Conor Gilligan, Kristen G. Anderson, Benjamin O. Ladd, Yun Ming Yong, Michael David

**Affiliations:** 1 University of Newcastle, School of Medicine and Public Health, University Drive Callaghan, Newcastle, NSW 2308 Australia; 2 Reed College, 3203 SE Woodstock Blvd., Portland, Oregon 97202 USA; 3 Department of Psychology, Washington State University, 14204 NE Salmon Creek Ave, Vancouver, WA 98686 Canada

**Keywords:** Standard drink, Surveys, Measures, Underestimation, Alcohol

## Abstract

**Background:**

Alcohol consumption estimates in public health predominantly rely on self-reported survey data which is likely to underestimate consumption volume. Surveys tend to ask specifically about standard drinks and provide a definition or guide in an effort to gather accurate estimates. This study aimed to investigate whether the inclusion of the term standard drinks with pictorial guide is associated with an adjustment in self-reported alcohol volume.

**Methods:**

A web-based survey was administered with AUDIT-C questions repeated at the beginning and end of the survey with and without the standard drink term and guide. The order in which respondents were presented with the different question types was randomised. Two cohorts of university/college students in NSW Australia (*n* = 122) and the US Pacific Northwest (*n* = 285) completed the survey online.

**Results:**

Australian students did not adjust their responses to questions with and without the standard drink term and pictorial guide. The US students were more likely to adjust their responses based on the detail of the question asked. Those US students who drank more frequently and in greater volume were less likely to adjust/apply a conversion to their consumption.

**Conclusions:**

This study supports previous findings of the inaccuracy of alcohol consumption volume in surveys, but also demonstrates that an assumption of underestimation cannot be applied to all individual reports of consumption. Using additional questions to better understand drink types and serving sizes is a potential approach to enable accurate calculation of underestimation in survey data.

## Introduction

Population-level estimates of alcohol-consumption and the level of risk associated with drinking rely on self-reported data defining consumption in terms of ‘standard drinks’. The standard drink measure was conceived to standardise the volume of pure alcohol in beverages served in commercial settings, but has since been used to quantify safe or low risk drinking levels [1] and alcohol consumption data collected in surveys [2].

International variations exist in the definition of a standard drink. Individuals’ understanding of, and ability to apply the concept to survey responses or self-poured servings of alcohol has been associated with an underestimation of consumption [3]. Studies consistently show that estimates of alcohol consumption based on self-reported surveys underestimate drinking volume, with some approaches accounting for as little as 40 to 60% of alcohol sales [4]. Other approaches, using location and beverage specific questions have been found to account for up to 94% of taxable alcohol [5, 6]. Such underestimation has important implications for the measurement of alcohol-related risk at both individual and population levels, and the interpretation of alcohol-related burden of disease data. A review of studies using tasks such as self-pouring and image selection, reported that over-estimation of standard drink size (pouring more than the volume of a standard drink) was common to participants in the US, UK, Australia, and the Netherlands, despite substantial differences in each countries’ definition [3]. It follows that responses to survey questions about standard drink consumption are likely to underestimate the actual volume consumed.

Several studies have reported differences in the volume of alcohol consumption reported in response to varied question styles including quantity-frequency, graduated-frequency, and recent recall [7–9]. While the Alcohol Use Disorders Identification Test (AUDIT-C) is a widely used and validated approach to screen for alcohol misuse [10, 11], its use as an estimate of consumption volume is problematic. Quantity-frequency questions, such as the AUDIT-C, typically use the mid-point for number of drinks and number of days in each response option to generate consumption volume (e.g. assuming 2.5 days per week for the option ‘2 or 3 days per week’), and underestimate consumption compared with a question about how many drinks were consumed on the previous day [12, 13]. In comparison to ‘yesterday’ methods however, quantity-frequency approaches are less likely to overestimate abstention, and have comparable criterion and predictive validity [14]. The AUDIT-C also uses a longer reference period of the last three months which is likely to be associated with greater recall bias than shorter periods such as three days or the last three or four drinking occasions [15, 16]. Stockwell et al. suggest that underestimation in surveys can be attributed to forcing respondents to report in terms of standard drinks and on typical days, not allowing for differences on weekends or different weekdays [8]. Importantly, underestimation of consumption is reported to be greater among younger male drinkers [13, 17], middle-aged female drinkers, and those who engaged less frequently in heavy episodic drinking, and is less marked among young and older females [6]. There also appears to be differential under-reporting of beer compared to other beverages [12]. Important differences also emerge depending on the way questions and response options are presented, with respondents tending to select one of the first few visible options in cases such a drop-down menu of options [9].

Part of the explanation for variance in reporting, and for under-reporting, may be the varied drinking patterns and socio-cultural norms in different drinking contexts and subcultures. Alcohol is consumed differently in private settings relative to commercial settings and licensed premises, although this variation is likely to be more relevant to some beverage types than others [3]. For example, beer is generally consumed in fixed serving sizes (which differ within and between countries), while wine is poured to various levels in various glass sizes [3, 18]. In licensed premises, there is little or no stipulation of the sizes or alcohol volume of beverages served. In Australia and the USA, liquor licensing requires serving staff to complete training (Responsible Service of Alcohol/ Service Permit), but the focus of training is knowledge of alcohol policies, identification of intoxicated patrons, and ensuring that intoxicated patrons are not served alcohol as opposed to ‘serving’ alcohol per se [19, 20]. The variation in consumption styles reinforces the need for standardised measures to quantify consumption in surveys.

Irrespective of whether a survey question asks about consumption ‘yesterday’ or ‘on a typical day’, population-level consumption volume estimated from brief survey questions, usually relies on the assumption that respondents are reporting standard drink consumption. Given the evidence for over-estimation of standard drink size [3], this typically requires an adjustment of responses from the number of drinks or serves an individual consumes to convert into standard drinks. Many surveys present a figure including images of typical beverage types and containers, indicating the number of standard drinks in each to aide this conversion or adjustment [21, 22], but the extent to which individuals ‘adjust’ their answers in response is unknown. Previous studies have predominantly compared alcohol consumption estimated with different cohorts [17, 23] or types of questions [12] but have not specifically measured the impact of the term ‘standard’ drink or the inclusion of a standard drinks guide.

### Aims

Here, we investigate whether the inclusion of the standard drink term and pictorial guide is associated with an adjustment in self-reported alcohol consumption among two cohorts of university/college students in New South Wales, Australia, and the US Pacific Northwest. The aims of the study were to: 1) establish the extent to which providing a definition of a standard drink led to within-subject differences in reporting of alcohol consumption; and 2) explore factors associated with differences in reporting of alcohol consumption. Both aims are considered from a cross-national perspective comparing samples from Australia and the USA in a convenience approach based on the researchers’ institutional affiliations and access to student populations. It is reasonable to expect differences in the cultural norms between the US and Australia given different tertiary education structures, as well as drinking, secondary supply and alcohol purchase laws.

## Methodology

### Participants - recruitment

Participants were recruited from one university in New South Wales (NSW), Australia, one in Washington, USA, and a college in Oregon, USA (Pacific Northwest sample). Recruitment materials invited students aged 18 years and above who drink alcohol to complete an anonymous, online survey.

In NSW, students were recruited using convenience, non-probability sampling through posts on the course management sites of courses across the university as well as through student networks such as residents groups. No compensation was offered for completion. Given the potential that content associated with alcohol consumption would be covered in some programs, participants were recruited from a range of faculties and programs. In Australia there is no legal drinking age per se, but the age for the legal purchase of alcohol is 18 years.

In Oregon, students at a small, liberal arts college were recruited via posters placed on campus and online posts. In Washington, students were recruited from two campuses of a large, public university. Students were recruited from the undergraduate psychology research pool including any student enrolled in a psychology course (not necessarily psychology majors). Students could select this study from a list and were compensated for completion via course credit. The legal drinking age in the USA is 21 years of age; however, illicit drinking is common among university students under the legal age of consumption [24].

### Measures

**Fig. 1:**
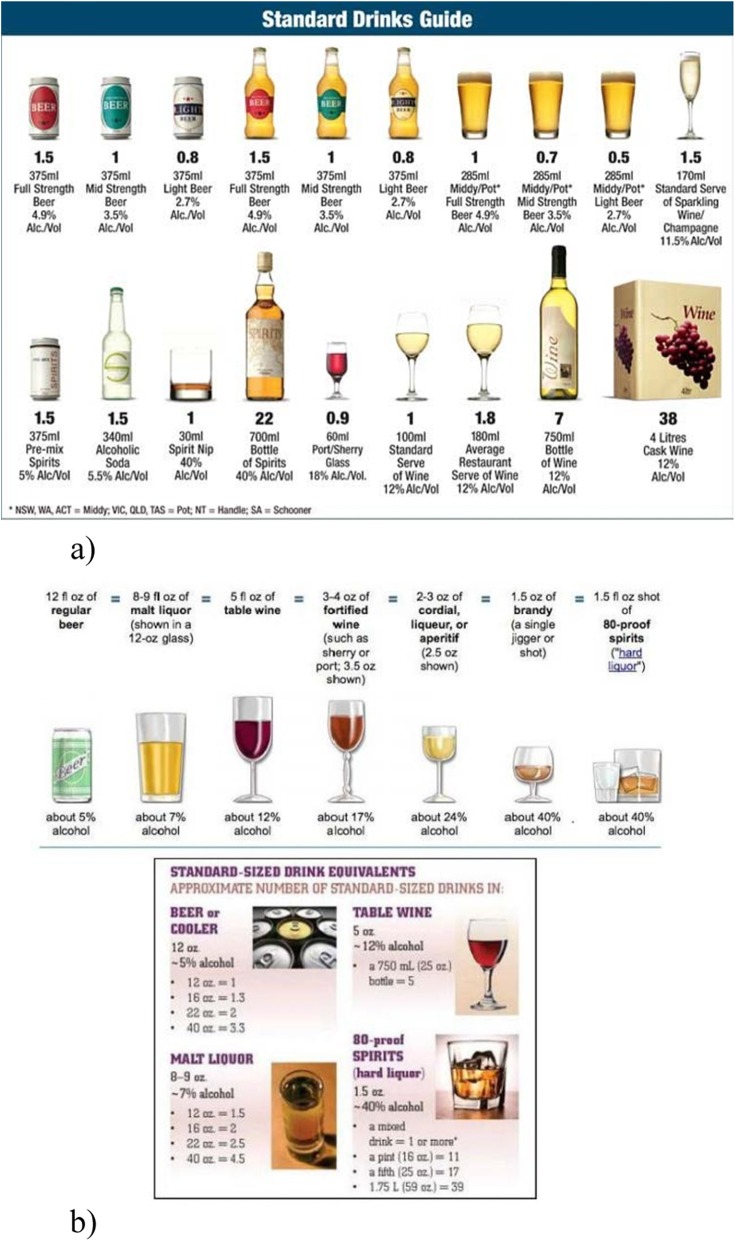
a) Australian Standard Drinks Guide used in survey (Australian Government Department of Health https://www.health.gov.au/health-topics/alcohol/). b) US Standard Drinks Guide used in survey (National Institute on Alcohol Abuse and Alcoholism https://www.rethinkingdrinking.niaaa.nih.gov/How-much-is-too-much/what-counts-as-a-drink/whats-A-Standard-drink.aspx, and Centers for Disease Control https://www.cdc.gov/ncbddd/fasd/women.html

Procedures and measures were identical for both cohorts apart from the use of country-specific standard drinks images. The survey contained three blocks of questions, with slightly different versions used in Australia and the US Pacific Northwest to account for different standard drinks definitions and drinking cultures. In Australia, the standard drink size is that which contains 10 g of pure alcohol, while the US equivalent is 14 g of pure alcohol. The first block contained three questions from the AUDIT-C (see https://www.integration.samhsa.gov/images/res/tool_auditc.pdf for a reference guide) accompanied by an Australian Standard Drinks Guide or equivalent US image (see Fig. 1 a) and b)). The three questions were (1) “How often do you have a drink containing alcohol,” (2) “How many standard drinks do you have on a typical day when drinking,” and (3) “How often do you have six or more standard drinks on one occasion.” A second block acted as a diffuser, and the final block utilised a modified version of the AUDIT-C (mAUDIT-C), which asked the AUDIT-C questions, but replaced the term ‘standard drink’ with either ‘drinks’ or ‘serves’ and removed the pictorial guide. Blocks one and three were randomised for each participant. 50.5% of respondents saw the term ‘serves’ and 49.5% saw the term drinks. Participants were not allowed to return to previous question blocks.

Alcohol consumption volume arising from the questions in this block was reported as an ordinal variable, with respondents reporting the number of [standard] drinks consumed on a typical day as 1 or 2, 3 or 4, 5 or 6, 7 to 9, or 10 or more. The mid-point of these responses was taken as the number of drinks reported (1.5, 3.5, 5.5, 8, 12). We created a dichotomous variable to measure adjustment of alcohol consumption volume based on whether the number of ‘standard’ drinks reported was significantly different from the number reported in response to questions about drinks or serves only. This measure was used as the dependent variable in logistic regression analyses.

The second block of questions included items about self-reported drinking status (non-, occasional-, light-, party/social-, heavy-, or ex-drinker), the type of alcohol usually consumed (with ten options as well as an ‘other’ option provided along with locally relevant examples, e.g. ordinary [full strength] beer, low alcohol beer, wine, wine coolers, champagne or sparkling wine, alcoholic cider, alcoholic sodas, premixed spirits, spirits, liqueurs including premixed liqueurs), the places where the respondent usually drinks (In my own/spouse’s/partner’s home, at a friend’s house, at a party at someone’s house, at raves/dance parties, at restaurants/cafés, at licensed premises (e.g. pubs, clubs), at University, at my workplace, in public places (e.g. parks, beaches), in a car or other vehicle, or other), and a series of items relating to drinking intentions (thinking of all the times that you drink alcohol, how often would you drink for the following reasons …) and alcohol-related harms (e.g., being sick after drinking, violence, unwanted sexual activity). These items were selected based on the likelihood they would be associated with reported volumes, and participants were able to select multiple options.

### Sample

One hundred twenty-two participants completed the survey in NSW and 285 in the Pacific Northwest USA. The participant demographics and differences between cohorts are summarised in Table 1. A majority of participants in both cohorts identified as female, the average age was 21 (20.98; SD = 3.47; Australian sample slightly older; *p* < 0.001), and most were in the first three to four years of an undergraduate program. The Australian sample were predominantly in the first two years of a program, while US students were more commonly in their third or fourth year (p < 0.001). Australian students were enrolled in 23 different programs, with Electrical Engineering, Oral Health, Pharmacy, and alternate entry programs most common. US students came from 28 different programs with Civil Engineering, Computer Systems Engineering, Professional Engineering, Energy Studies, or Linguistics most common.

**Table 1:** Characteristics of students who self-reported as drinking alcohol in the past year

Characteristics, N (%) or median (IQR)	Total	Australia	USA	*P*-value
	*N* = 407(%)	*N* = 122 (%)	*N* = 285 (%)	
Gender^a^				0.127^j^
Male	103 (25.31)	37 (30.33)	66 (23.16)	
Female	304 (74.69)	85 (69.67)	219 (76.84)	
Year of Program^b^				< 0.001^k^
1st Year	127 (31.28)	54 (44.26)	73 (25.7)	
2nd Year	75 (18.47)	30 (24.59)	45 (15.85)	
3rd Year	113 (27.83)	29 (23.77)	84 (29.58)	
4th Year +	91 (22.41)	9 (7.38)	82 (28.87)	
Program Type^c^				< 0.001^k^
Undergraduate	401 (98.53)	116 (95.08)	285 (100.00)	
Postgraduate	6 (1.47)	6 (4.92)	0 (0.00)	
Type of Drinker^d^				0.005^k^
Occasional drinker	108 (26.54)	41 (33.61)	67 (23.51)	
Light drinker	29 (7.13)	13 (10.66)	16 (5.61)	
Party/Social drinker	259 (63.64)	63 (51.54)	196 (68.77)	
Heavy drinker	9 (2.21)	5 (4.10)	4 (1.40)	
Ex-drinker	2 (0.49)	0 (0.00)	2 (0.70)	
Age^e^ - mean (SD)	20.98 (3.47)	22.25 (4.25)	20.43 (2.92)	< 0.001^l^
Age of first drink^f^ – mean (SD)	16 .37 (1.82)	16.24 (1.76)	16.43 (1.82)	0.346^l^
AUDIT-C^g^ - mean (SD)	4.53 (2.78)	4.43 (2.78)	4.58 (2.04)	0.544^l^
Type of drink^h^				
Full strength beer	146 (35.87)	38 (31.15)	108 (37.89)	0.215^k^
Low strength beer or Wine-cooler	66 (16.22)	5 (4.10)	61 (21.4)	< 0.001^k^
Wine	143 (35.14)	61 (50.00)	82 (28.77)	< 0.001^k^
Champagne	61 (14.99)	27 (22.13)	34 (11.93)	0.010^k^
Cider	102 (25.06)	52 (42.62)	50 (17.54)	< 0.001^k^
Soda or Premix	139 (34.15)	43 (35.25)	96 (33.68)	0.820^k^
Spirits	228 (56.02)	63 (51.64)	165 (57.89)	0.276^k^
Liquers	49 (12.04)	14 (11.48)	35 (12.28)	0.870^k^
Other	8 (91.97)	3 (2.46)	5 (1.75)	0.701^k^
Location where drink consumed^i^				
Home	188 (46.19)	188 (59.84)	115 (40.35)	< 0.001^k^
Friend’s place	223 (54.79)	72 (59.02)	151 (52.98)	0.278^k^
House party	247 (60.69)	75 (61.48)	172 (60.35)	0.912^k^
Rave party	95 (23.34)	30 (24.59)	65 (22.81)	0.703^k^
Cafe	89 (21.87)	43 (35.25)	46 (16.14)	< 0.001^k^
Licensed premises	157 (38.57)	89 (72.95)	68 (23.86)	< 0.001^k^
University	116 (28.50)	16 (13.11)	100 (35.09)	< 0.001^k^
Workplace	9 (2.21)	5 (4.10)	4 (1.40)	0.135^k^
Public place	24 (5.90)	7 (5.74)	17 (5.96)	1.000^k^
Car	10 (2.46)	2 (1.64)	8 (2.81)	0.730^k^
Other	5 (1.23)	2 (1.64)	3 (1.05)	0.638^k^

^a^n(Gender) = 407; ^b^n(Year of Program) = 406; ^c^n(Program Type) = 407; ^d^n(Type of Drinker) = 407; ^e^n(Age) = 407; ^f^n(Drinking Age Commencement) = 407; ^g^n(AUDIT-C) = 405; ^h^n(Type of drink) = 407; ^i^n(Location where Drink consumed) = 407; ^j^Pearson’s Chi-squared test; ^k^Fisher’s exact test; ^l^Mann-Whitney U-test; ^jl^Independent t-test

In both cohorts, participants most commonly referred to themselves as party/social drinkers (51.5 and 68.8% of Australian and US cohorts, respectively), or occasional drinkers (33.6 and 23.5% of Australian and US cohorts, respectively). Age of initiation to drinking ranged from 9 to 21 years (mean 16.67; SD 1.82) and was not significantly different between cohorts. The types of drinks ‘usually’ consumed were spirits (56.0%), full strength beer (35.9%), wine or champagne (50.1%), pre-mixed drinks/alcoholic sodas (34.15%), and cider (25.1%) with differences between the cohorts in beverage selection (see Table 1). The most commonly identified locations of drinking were home/friend’s home/house party followed by licensed premises, rave parties, university, and cafes.

The average AUDIT-C score (based on the validated instrument using the standard drink term and image) was 4.53 and did not differ significantly between the groups. Forty-three percent of Australian and 44% of US respondents scored above 4 on the AUDIT-C, indicating possible hazardous drinking. In both samples, males were more likely to score above 4 than females (Australia male:female = 54.05%: 51.81%; US male:female = 68.18%: 60.27%). For a small number of women (*n* = 6 Australian and *n* = 4 US), all four points were from the first question which is suggestive of lower risk drinking.

### Analysis

Continuous variables were reported as mean (SD), and categorical variables as counts (%). Continuous variables were compared by means of the two-sample Student’s t-test for independent samples with equal and unequal variances (as appropriate), and ordinal variables were compared using the Pearson Chi-squared test and the Fisher’s Exact test (as appropriate). Paired data were compared using the McNemar-Bowker test. Due to the cross-over design, the effects of carryover, period and sequence were assessed and found to be non-significant. The analysis of response adjustments was modelled using Firth logistic regression, without the presence of these effects. Separate models for Australian and USA students were built using a two-stage process. First, univariate analyses were performed to determine the strength of association between independent variables and adjustment of drinking volume. Independent variables included gender, age, number of standard drinks consumed on a typical drinking day, frequency of consumption, frequency of consuming more than six standard drinks, type of alcohol consumed, places where alcohol is typically consumed, alcohol-related harms and alcohol expectancies. Second, variables with a *p*-value < 0.10 in the univariate analyses were included in the multivariable model. Firth’s logistic regression analysis was used to overcome the computational limitations and convergence issues caused by the sparseness (separation of the data [25]). Model validity was evaluated by performing the Hosmer-Lemeshow goodness-of-fit test and the Pregibon link test [26] on each model. A *p*-value< 0.05 by the two-tailed test was considered to be statistically significant. All analyses were performed using Stata Version 15 (StataCorp LP, College Station, TX). Post-hoc analysis was performed with the US sample age-stratified to assess any potential differences between under-age respondents and those legally able to purchase and consume alcohol.

## Results

### Alcohol consumption volume

Differences in reporting of alcohol consumption associated with the presentation of the term and guide for standard drinks were assessed. In the Australian sample, participants reported consuming an average of 5.41 (SD = 3.49) ‘standard’ drinks on a typical drinking day, and reports for drinks and serves were 5.16 (SD = 3.46) and 5.76 (SD = 3.84) respectively. In the US sample, the reported number of standard drinks was 6.58 (SD = 1.95), drinks was 4.75 (SD = 2.13), and serves was 4.65 (SD = 2.23). There were no statistically significant differences between reports of drinks or serves in either sample, so these were grouped in analysis. When reported standard drinks were compared with reported drinks/serves combined, there was almost no difference in the Australian sample (0.01, 95% CI: − 0.30 to 0.31, *p* = 0.977) while the values were significantly different in the US sample (1.88, 95% CI: 1.58 to 2.17, *p* < 0.001; Table 2). Differences were maintained in the US sample stratified for age (< 21 years versus 21 and over).

**Table 2:** Comparison between the number of standard drinks on a typical day with the number of drinks/serves for Australian and USA students

	Standard drinks	Drinks/Serves	*P*-value
Australia^a^	5.41 (3.49)	5.40 (3.49)	0.977^c^
US^b^	6.58 (1.95)	4.70 (2.18)	< 0.001^c^
US < 21 yrs	6.52 (1.83)	4.87 (2.16)	< 0.001 ^c^
US 21+ yrs	6.66 (2.13)	4.44 (2.19)	< 0.001 ^c^

^a^113 Australian students reported number of standard drinks and number of drinks/serves (i.e. 9 cases excluded from analysis due to one or more missing values); ^b^285 US students (174 < 21 years and 111>/=21 years) reported number of stand drinks and number of drinks/serves; ^c^Independent t-test

### Alcohol consumption frequency

Frequency of consumption differed for reports of standard drinks, and drinks/serves across the entire sample (Table 3) No significant differences were found between reports of drinks and serves, so these were grouped. For standard drinks and drinks/serves, frequency also differed between the cohorts. Australian students reported more frequent consumption of standard drinks, with fewer reporting drinking monthly or less (25.0% vs 53.0%), and more drinking 2–4 times per month (40.8% vs 31.2%), 2 or 3 times per week (25.8% vs 12.3%), and 4 or more times week (8.3% vs 3.5%) compared to US students. Frequency of drinking six or more standard drinks, drinks, or serves did not differ significantly between cohorts or terms (not shown). Differences in frequency reports were statistically significant when comparing standard drinks and drinks/serves for US students (*p* < 0.001) but not for Australian students (*p* = 0.083). In the US sample, this difference was larger among students who were under 21 years of age, compared to ‘of age’ drinkers (*p* < 0.0001; not shown).

**Table 3:** Frequency of students having at least one standard drink/drink/serve in the past year

	Standard drinks N (%)	Drinks/Serves N (%)	P-value^j^
Frequency of drinking			
All students^a, b, c^			
Monthly or less	181 (44.69)	71 (17.75)	
2–4 times per month	138 (34.07)	162 (40.50)	
2or 3 times per week	66 (16.30)	149 (37.25)	
4+ times per week	20 (4.94)	18 (4.94)	< 0.001
Australian students^d, e, f^			
Monthly or less	30 (25.00)	33 (28.70)	
2–4 times per month	49 (40.83)	41 (35.65)	
2or 3 times per week	31 (25.83)	34 (29.57)	
4+ times per week	10 (8.33)	7 (6.09)	0.083
USA students^g, h, i^			
Monthly or less	151 (52.98)	38 (13.33)	
2–4 times per month	89 (31.23)	121 (42.46)	
2or 3 times per week	35 (12.28)	115 (40.35)	
4+ times per week	10 (3.51)	11 (3.86)	< 0.001

^a^n(All students who reported drinking standard drinks) = 405; ^b^n(All students who reported drinking drinks or serves) = 400;

^c^n(Australian students who reported drinking standard drinks and drinks or serves) = 398; ^d^n(Australian students who reported drinking standard drinks) = 120;

^e^n(Australian students who reported drinking drinks or serves) = 115; ^f^n(Australian students who reported drinking standard drinks and drinks or serves) = 113;

^g^n(USA students who reported drinking standard drinks) = 285; ^h^n(USA students who reported drinking drinks or serves) = 285; ^ii^n(USA students who reported drinking standard drinks and drinks or serves) = 285; ^j^Stuart-Maxwell test for marginal homogeneity

### Factors associated with lack of adjustment

Ninety-one respondents (80.5% of valid responses) in the Australian sample and 63 respondents (22.1%) in the US sample made no adjustment to their responses of typical number of drinks consumed. The difference in adjustment between age groups in the stratified US sample was not significant, though there was a trend towards smaller adjustment among those aged less than 21 (Table 2). In the Australian sample, multivariable logistic regression of factors potentially associated with a lack of adjustment found that there was no association between age, gender, number of standard drinks/drinks/serves on a typical day, frequency of drinking, or frequency of consuming six or more drinks and the probability of adjustment (Table 4). Further, none of the secondary predictors (types of drinks consumed, reasons for drinking, harms associated with drinking and the locations of drinking) were significantly associated with adjustment at the univariate level and were therefore excluded from the regression model. The model with primary predictors only was found to have good fit (*p* = 0.675) and be appropriately specified (*p* = 0.846).

**Table 4:** Multivariable Firth logistic regression analysis predicting adjustment of typical drink quantity based on survey format

Variable	Australia				US			
	OR	95% CI	*P*-value	Overall *p*-value	OR	95% CI	*P*-value	Overall *p*-value
Age	0.98	0.86–1.13	0.88		1.05	0.95–1.15	0.33	0.33
Gender (Ref: Male)	1				1			
Female	0.90	0.22–3.70	0.82		0.77	0.39–1.53	0.46	0.46
Number of drinks on a typical day (Ref: 1 or 2)	1			0.46	1			< 0.01
3 or 4	0.17	0.01–3.77	0.26		3.11	0.16–60.94	0.46	
5 or 6	0.18	0.01–5.18	0.32		13.51	0.71–256.61	0.08	
7 to 9	0.10	0–3.23	0.19		5.98	0.30–118.38	0.24	
10+	0.91	0.01–72.63	0.72		8.03	0.32–202.61	0.21	
Frequency of drinking in the past 3 months (Ref: Monthly or less)	1			0.71	1			< 0.01
2 to 4 times a month	2.20	0.27–17.97	0.46		4.08	1.81–9.20	< 0.01	
2 or 3 times a week	0.86	0.06–12.48	0.91		1.54	0.56–4.24	0.40	
4+ times a week	0.52	0.01–18.01	0.72		3.06	0.71–13.14	0.13	
Frequency of drinking 6+ drinks on one occasion (Ref; Less than monthly)	1			0.29	1			0.28
Monthly	0.29	0.04–2.27	0.24		0.91	0.40–2.07	0.83	
Weekly	1.27	0.07–24.54	0.87		2.27	0.69–7.44	0.18	
Daily or almost daily	0.25	0–30.14	0.57		0.71	0.02–28.70	0.86	
In the past 12 months planned to get drunk before drinking (Ref: Never)					1			0.02
Once					0.10	0.01–0.57	0.01	
Twice					0.54	0.13–2.31	0.41	
3–5 times					0.19	0.06–0.62	0.01	
5–11 times					0.14	0.04–0.49	< 0.01	
12+ times					0.21	0.06–0.71	0.01	

In the US model (Table 4), significant overall effects were found for the number of drinks/serves/standard drinks on a typical day (*p* = 0.003) and the frequency of drinking in past 3 months (*p* = 0.003) in predicting adjustment of survey response. Those who reported drinking more in terms of both frequency and volume were less likely to adjust their consumption. Having planned to get drunk was found to have a significant positive association with the probability of adjustment (*p* = 0.02). The model had good fit (*p* = 0.092) and was appropriately specified (*p* = 0.365.)

## Discussion

The results suggest that Australian students in this study do not adjust/convert their survey responses to account for standard drink units. Among the US participants, however, responses differed when asked about standard drinks as opposed to drinks or serves, suggesting that adjustment was occurring to account for the standard drinks guide presented. A lack of adjustment is interpreted here as likely reflecting an underestimation of consumption, given previous evidence of over-estimation of standard drink size [3]. In the absence of a gold-standard measure, however, it cannot be guaranteed that this is the case, with other possible explanations including greater consistency of reporting, greater awareness of the standard drink size, or actual serves which more closely reflect standard drink sizes among Australian respondents.

These findings are partly in keeping with previous literature from standard drink perception studies [3] and studies validating survey estimates [13]. The present study suggests that an assumption of under-estimation should not be applied to all population groups, or indeed, to entire datasets. The difference between cohorts in this study is an unexpected finding, suggesting that some cohort effects or national differences are likely to influence an individual’s tendency to adjust their responses.

The adjustment performed by US students could reflect a greater understanding of the standard drink concept, possibly due to relevant learning in their programs of study or college orientation, an assumption that the ‘repeated’ questions warranted different responses, or a more pronounced difference between actual serving sizes and standard drinks. Alternatively, it is possible that this group had less understanding of the standard drink concept prior to study participation, hence greater adjustment upon presentation of the standard drink definition, but in that case it is difficult to speculate as to the basis of the difference between responses. While there is evidence for a lack of knowledge of the amount of alcohol contained in standard drinks [27] among the Australian population, there is a lack of evidence for the effectiveness of standard drinks guides to improve individual’s knowledge and modify estimates. An Australian national survey found very poor knowledge of guidelines for low-risk of short- and long-term harm from drinking, with less than 5% of respondents able to accurately identify the levels [23].

The pattern of volume adjustment occurring in the US but not the Australian sample is also reflected in the frequency of drinking. It would be expected that the frequency of alcohol consumption would remain the same, regardless of adjusted volumes. This pattern could reflect a misunderstanding of the nuances between the questions. It is possible that students in the US sample interpret the question about the frequency of drinking ‘standard drinks’ as a question about drinking in a certain way or a certain type of drink (as displayed in the pictorial guide) rather than as the frequency of drinking per se. The interpretation of the standard drink term may differ among different population subgroups according to drinking style or age. While age-based differences were not significant in this cohort, there was a pattern in which underage US drinkers reported slightly less standard drink consumption but slightly more drink/serve consumption than their older counterparts. This supports a need to carefully interpret survey data with different sub-groups, with indications that different groups may interpret survey questions differently.

National differences might to some extent, explain the difference between the cohorts, with subtle but important variations between student subcultures. The gender imbalance was more pronounced in the US sample, with 76.8% of the US respondents identifying as female compared to 69.7% of the Australian sample. While this difference was not statistically significant, the dominance of females may have influenced the results, particularly given established differential under-reporting between age and gender groups [6]. Other important differences between the samples include age and year of study, with more US students in the third year or higher of their degree. This could be reflective of greater maturity, advanced education about the risks of drinking and standard drink guides, or of students having ‘grown out’ of the heavy drinking culture often associated with the early university/college years. On the other hand, Australian students had more years of drinking including years of legal drinking, thus it is possible that drinking experience accounts for higher rates of social and heavy drinking in this group. Heavy drinking cultures are often associated with freshman and early college or university years. Studies in the US have demonstrated that patterns of drinking change over the college years, with some evidence for a reduced prevalence of heavy drinking with advancing years [28, 29]. The present data fit this pattern, with a larger proportion of underage drinkers in the US reporting consuming six or more drinks monthly or weekly (47.1%) compared to those aged 21 and over (37.8%).

National differences between the samples are also suggested in the variation of drink types selected. Consumption of wine, champagne, and alcoholic cider was more commonly reported by the Australian students compared to those in the US Pacific Northwest, who more commonly reported drinking low strength beer or wine coolers. Further, the locations of consumption differed with more Australian students reporting drinking at home, in cafes, or in licensed premises. The latter is likely to be a product of the fact that in the US, students are not legally allowed to drink alcohol until the age of 21, and therefore are less likely to drink in public settings. Previous studies have investigated the volume of alcohol consumed in an individual serving in different settings, with wine served and poured by women in both public and private settings likely to be in excess of a standard drink [18], while serves of beer or fortified wine poured in private settings may be less than a standard drink [18, 30].

In the US sample, adjustment appears to occur less frequently at higher levels of drinking. This is consistent with findings that heavy drinkers may under-report their alcohol use to a greater extent than lighter drinkers [31] and is in keeping with Australian findings suggesting that high risk drinkers are less likely to accurately estimate low risk drinking levels [32]. In contrast, however, the same study reported that individuals who had seen a standard drinks logo were more likely to accurately estimate levels of drinking that reduce the risk of long-term harms [32], and other research has suggested that those who infrequently or never engage in heavy episodic drinking underestimate their drinking by larger proportions than those who do [6]. Therefore, some drinking experience and exposure to drinks and labelling might be expected to educate drinkers about alcohol volume.

The study was limited by a small sample of voluntary participants, dominated by females. It is not possible to estimate the recruitment rate due to the nature of recruitment, but it is expected that the response rate was low. As such, the sample may not be representative of either student population. Further, it is likely that the female dominance of the sample translates into a different pattern of beverage types, volume, and frequency. Also, the recruitment of university students, while useful in targeting a group of high-risk drinkers, may bias the sample towards well-educated respondents likely to be better placed than the average population to understand the standard drink concept and associated adjustments.

Answers to volume and frequency questions were non-specific to the types of alcohol and contexts in which it is consumed. Participants were able to select multiple responses to the questions about alcohol type and location. It is likely that alcohol consumed at parties is different in both type and volume to that consumed at home, a friend’s house, or at a café. Further, variation exists within each beverage category. For example, the emerging popularity of craft beers which are typically high alcohol volume, and the marketing of ‘healthier’, ‘lighter’ wine options complicates the estimate of actual alcohol volume consumed. The survey did not enable the selection of these beverage alternatives specifically. Without detailed mapping of consumption according to each of these parameters, likely underestimation cannot be completely understood. While this study does not provide a representative cultural comparison between these two countries, the data offers a snapshot of survey response behaviours which warrants consideration in future studies.

Internationally, we rely on surveys to monitor trends and provide an indication of alcohol-related health risk and burden. However, while surveys might capture trends in per capita consumption they do not necessarily provide an accurate estimate of volume [33]. It will therefore be important for future monitoring by public health agencies and policy-makers to better understand the discrepancies between reported and actual consumption.

## Conclusions

This study supports previous findings of underestimation of alcohol consumption volume in population surveys, but demonstrates that the underestimation assumption should not be applied consistently to all individual reports of consumption. Further detail may be required in questions about drink type and serving size to more accurately quantify consumption. Location-and beverage-specific approaches which negate the need for a standard drink guide may outperform instruments such as the AUDIT-C in estimating volume, but may be less useful for assessments of risk. Adjustments made to frequency responses indicate that the standard drink term has more implications for respondents than merely the volume of consumption and raises important questions about the interpretation of standard survey questions such as the AUDIT-C. It appears that different population groups and potentially, age-based sub-groups interpret and respond to survey questions differently. Further research should explore the differing reports of consumption associated with different age groups, beverage types and contexts of consumption, as well as differences in responses to varied questions, in order to design survey questions which more accurately capture alcohol consumption volume and frequency.

## Data Availability

The datasets used and/or analysed during the current study are available from the corresponding author on reasonable request.

## Abbreviations

AUDIT-C: Alcohol Use Disorders Identification Test (Shortened Form)
IQR: Interquartile Range
SD: Standard Deviation
US(A): United States (of America)
